# Whole exome sequencing reveals a stop-gain mutation of PKD2 in an autosomal dominant polycystic kidney disease family complicated with aortic dissection

**DOI:** 10.1186/s12881-018-0536-6

**Published:** 2018-01-30

**Authors:** Wenwen Zhang, Qian Han, Zhao Liu, Wei Zhou, Qing Cao, Weimin Zhou

**Affiliations:** 1 Department of Vascular Surgery, The Second Affiliated Hospital of Nanchang University, No 1#, Minde Road, Nanchang, Jiangxi 330006 China; 2 Key Laboratory of Molecular Medicine of Jiangxi Province, Nanchang, Jiangxi China; 3 Department of Vascular Surgery, Nanjing Drum Tower Hospital, The Affiliated Hospital of Nanjing University Medical School, Nanjing, Jiangsu China

**Keywords:** Autosomal dominant polycystic kidney disease, Aortic dissection, *PKD2* mutation, Whole exome sequencing

## Abstract

**Background:**

Autosomal dominant polycystic kidney disease (ADPKD) is the most common inherited kidney disorder characterized by progressive cyst formation and expansion in the kidneys, which culminates in end-stage renal disease. Aortic dissection is a rare vascular complication of ADPKD and related literature is currently limited.

**Case presentation:**

In this report, we described a patient with asymptomatic Stanford B aortic dissection. Further investigation revealed a positive family history of ADPKD and normal renal function. Whole exome sequencing identified a stop-gain mutation c.1774C > T, p.Arg592Ter in the *PKD2* gene that segregated in the family. To our knowledge, this is the first report of ADPKD complicated with aortic dissection caused by *PKD2* mutation.

**Conclusions:**

The case illustrates the importance of aorta imaging and molecular diagnosis in ADPKD patients in order to achieve early recognition of the deadly vascular complication.

## Background

Aortic dissection is a potentially serious disease that in many cases can result in sudden death, if not given appropriate management. It is therefore crucial to identify such patients at early stage. Some patients develop aortic dissection as a result of congenital genetic aberration, and family clustering studies have suggested that at least 20% of aortic dissection patients have a first-degree affected relative [1]. With a better understanding of the relationship between genetic mutation and clinical course concerning aortic dissection, characterization of the underlying molecular defect might aid in providing prophylactic measures, genetic counseling and gene-based therapy [2].

The disease-causing genes of aortic dissection could be mainly categorized into three groups: components of extracellular matrix (*FBN1, COL3A1, MFAP5*), TGF-β signaling members (*TGFBR1, TGFBR2, SMAD3, TGFB2, TGFB3*) and contractile apparatus of smooth muscle cells (*ACTA2, MYH11, MYLK*) [3]. When these genes mutated, aorta phenotype is always present while extra-aortic features vary depending on penetrance. However, aortic dissection could also manifest as an uncommon trait in some monogenic disorders, for example, autosomal dominant polycystic kidney disease (ADPKD). ADPKD is the most common inherited nephropathy characterized by progressive cyst formation and expansion in the kidneys, which culminates in end-stage renal disease (ESRD) [4]. The majority of ADPKD cases are caused by mutations in either *PKD1* or *PKD2*. Vascular complications sometimes develop in patients with ADPKD. The most common form is cerebral aneurysms, whereas only a few cases of ADPKD-associated aortic dissection have been reported previously [5]. Among them, the underlying genetic aberration is rarely explored, failing to provide direct connection between aortic dissection and *PKD1/2* mutation [6].

Here we report the case of asymptomatic thoracic aortic dissection accompanied by ADPKD. By performing whole exome sequencing (WES), we identified a stop-gain mutation c.1774C > T, p.Arg592Ter in the *PKD2* gene that segregated in this family. The case underscores the importance of aorta imaging and molecular diagnosis in ADPKD patients in order to achieve early recognition of the deadly vascular complication.

## Case presentation

**Fig. 1:**
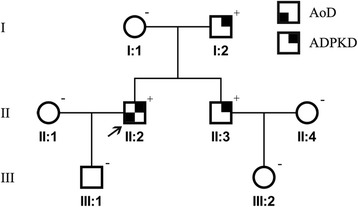
Pedigree; Proband is indicated with an arrow. Plus and minus sign indicate presence or absence of a PKD2 mutation, respectively. AoD: aortic dissection; ADPKD: autosomal dominant polycystic kidney disease

**Fig. 2:**
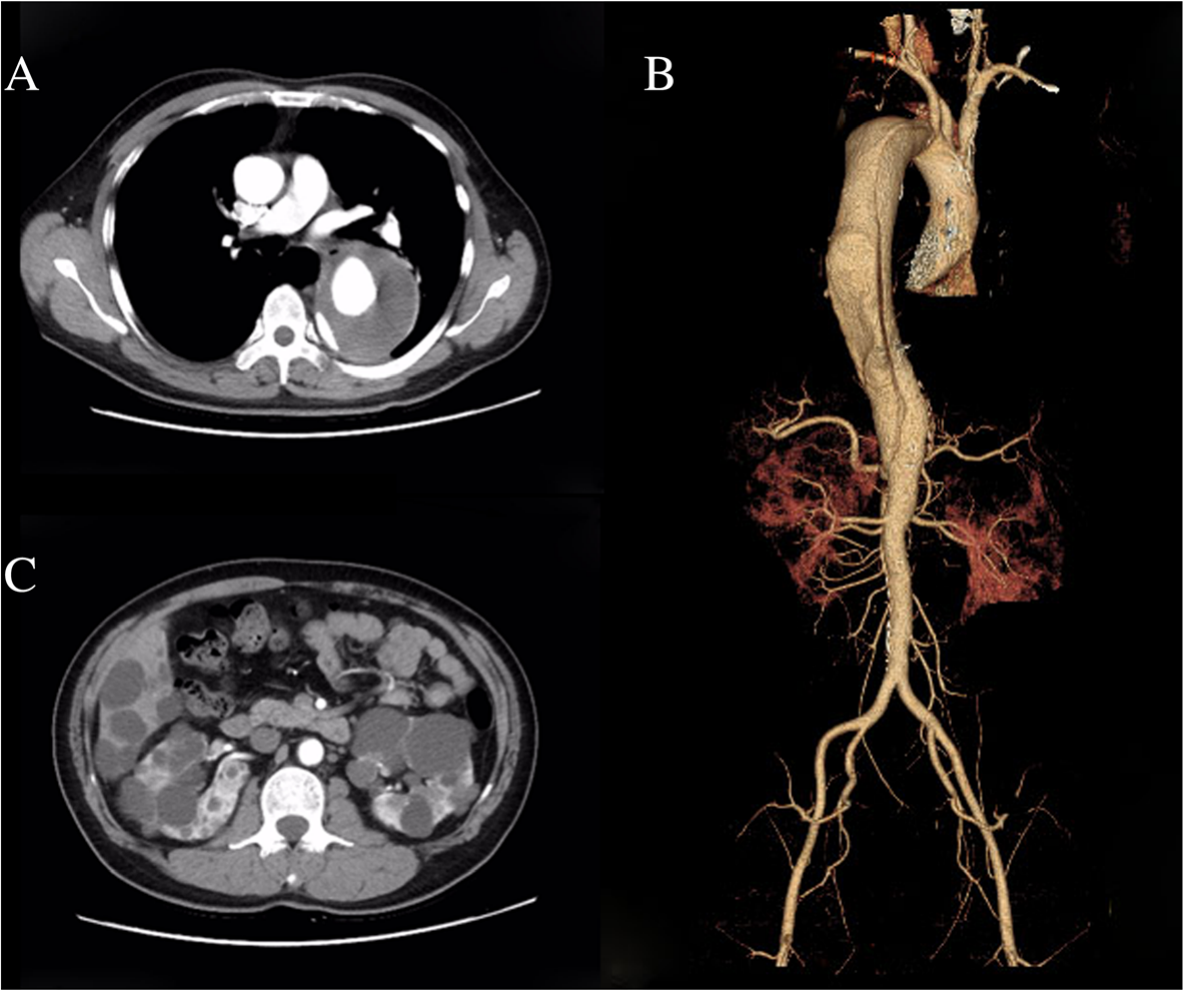
Radiographic findings of the proband. **a** Multi-slice computed tomography shows dissection aneurysm measuring 8.5 cm in diameter. **b** 3D–reconstructed computed tomography angiogram shows a Stanford B aortic dissection. **c** Multi-slice computed tomography shows multiple cysts in the liver and bilateral polycystic kidneys

The proband (Fig. 1, individual II:2), a 44-year-old male, was referred to our department complaining of abnormal enlargement of thoracic aorta detected in medical examination. His medical history was significant for hypertension for 10 years and blood pressure was 200/140 mmHg when admission. There is no history of acute chest and back pain in him. The patient underwent chest and abdominal enhanced computed tomography, revealing a dissection aneurysm measuring 8.5 cm in diameter (Fig. 2a). Three-dimension reconstruction showed a Stanford B aortic dissection originating immediately distal to the origin of the left subclavian artery, extending to the level of celiac trunk (Fig. 2b). He thereafter received strict blood pressure control therapy with intravenous sodium nitroprusside, which is converted to oral antihypertensive drugs captopril and betaloc. After the hemodynamic is stable, the patient was evaluated and then treated by endovascular stent-graft placement. The procedure was successful and he was discharged several days later.

Multiple variable-sized cysts in the liver and both kidneys were observed in his CT images suggestive for ADPKD (Fig. 2c). The patient’s serum creatinine was 79 umol/L (normal range, 44–106 umol/L). His family history was positive for ADPKD in the father and brother (individuals I:2 and II:3, aged 71 and 38 years old respectively), while their serum creatinine level was within normal limit (83 and 59 umol/L, respectively).

To understand the genetic basis of this family, whole exome sequencing was carried out on the proband. Genome DNA was extracted from peripheral blood using standard protocols and subjected to the exome capture procedure with the Agilent SureSelect Human All Exon kit (V5, 50 Mb) according to the manufacturer’s instruction. Paired-end sequencing was performed on an Illumina HiSeq 2500, and the short reads (150 bp) were aligned to the Human Genome (UCSC hg19) by Burrows-Wheeler Aligner (BWA). Raw image analyses and base calling were performed using Illumina’s Pipeline v.1.34 with default parameters. The Single Nucleotide Variants (SNVs) and small insertions and deletions (Indels) were generated with Genome Analysis Toolkit (GATK) and in parallel with the SAMtools pipeline. The called SNVs and Indels were annotated with ANNOVAR.

The variants were then filtered using standard methods, focusing on rare (minimal allele frequency < 0.01 in all populations from the Exome Sequencing Program and Exome Aggregation Consortium) and pathogenic (predicted deleterious by SIFT or PolyPhen2) variants. Sanger sequencing was performed on DNA samples of the proband and other family members for verification of the detected mutation and segregation analysis.

 On average 82 million reads were generated, among which, 99.7% were mapped to the human genome. The mean sequencing depth was 147-fold, and an average of 98.6% sequences was covered by more than 20-fold. With the data analysis and stepwise variants filtering strategy described previously [7], a heterozygous mutation c.1774C > T (NM_000297.3), p.Arg592Ter in the *PKD2* gene was identified. The mutation is predicted to encode a truncated product or result in nonsense-mediated decay of the transcript. Additionally, it was not present in Exome Sequencing Project, 1000 Genomes Project, Exome Aggregation Consortium or our in-house dataset.

**Fig.3:**
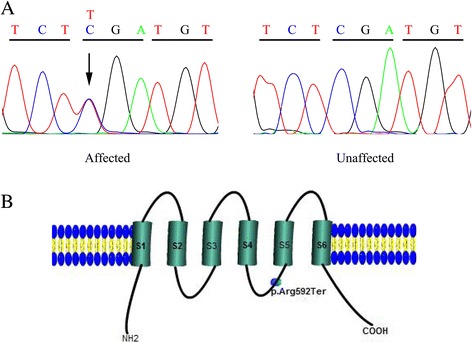
**a** The DNA sequencing chromatograms represent the affected and unaffected individuals. The corresponding DNA sequence is shown above the chromatograms and the arrow denotes the mutated nucleotide. **b** A graphic illustration of the 6 subunit transmembrane spanning PKD2 protein shows the position of identified mutation p.Arg592Ter

Further segregation analysis using Sanger sequencing revealed the same mutation in the proband’s affected father and brother (individuals I:2 and II:3), whereas the remaining members of the family (individuals I:1, II:1, II:4, III:1, III:2) were found negative (Fig. 3a). *PKD2* mutation-negative individuals underwent abdominal ultrasound examinations, excluding the existence of multiple cysts of liver and kidneys. All family members, including individuals II:1 and II:4, were subjected to enhanced computed tomography and no aortic lesions were found except the proband, suggesting low penetrance of the *PKD2* variant found in this family for aortic dissection.

## Discussion and conclusions

ADPKD is a common genetic disease characterized by multiple cysts formation in both kidneys and progressive loss of renal function. Mutations in either *PKD1* or *PKD2* are responsible for most cases of the disorder. Its encoded protein, polycystin, is involved in calcium transport and signaling in renal epithelial cells [8]. Previous studies have also demonstrated that polycystin is expressed in the smooth muscle cell and endothelial cell of the blood vessel, and plays a pivotal role in maintaining vascular integrity [9, 10]. Consequently, ADPKD patients are at an increased risk of developing intracranial aneurysms and aortic dissection [5]. However, as depicted in our case, thoracic aortic dissection is a rare vascular complication of ADPKD. This explains the scarcity of reports concerning ADPKD complicated with aortic dissection, leading to a lack of recognition by physicians [6]. Moreover, genotype-phenotype correlation regarding ADPKD-associated aortic dissection could hardly be established, making the identification of at-risk patients challenging. Silverio et al. summarized clinical features of ADPKD-associated aortic dissection patients and found that they were markedly younger than those in IRAD (International Registry of acute Aortic Dissection), despite a small sample number of patients [6]. More relevant studies are warranted to decipher the precise mechanism by which specific PKD mutations predispose to a vascular phenotype. ADPKD-associated vascular complication has become a life-threating condition and the predominant determinant of mortality. Unlike most aortic dissection patients, the index patient in our case had no episode of chest and back pain. Fortunately, he underwent a healthy checkup and a large dissection aneurysm was found. Our case emphasized the importance of aorta imaging in ADPKD patients with uncontrolled hypertension to exclude aortic diseases. Transesophageal echocardiography is a useful screening method, but enhanced computed tomography is recommended when aortic dissection is strongly suspected.

The *PKD2* gene extends over 70 kb of the genome and contains 15 exons that produce a 3 kb mRNA [11]. Its encoding product, polycystin-2, consists of 968 amino acids, which form six transmembrane domains [12]. Polycystin-2 interacts with polycystin-1 through its C-terminal domain, and this interaction is considered to be indispensable for fluid-flow sensation by the primary cilium of renal epithelial cells [8]. The *PKD2* mutation (R592X) identified in our study introduces a premature stop codon that leads to removal of the last 377 amino acids, altering both the transmembrane segments and the coiled-coil region located in the C-terminus (Fig. 3b). As a result, the mutation could impair protein function and hence was considered pathogenic. Aligned with this, a recent study had demonstrated that upregulation of polycystin-2 expression in mice significantly alleviated ADPKD phenotype in a dose-dependent manner [13]. The mutation was segregated in the family and had been previously reported in one study, which screened *PKD1* and *PKD2* mutations in a large cohort of ADPKD patients [14].Case reports of dissection aneurysm in patients with ADPKD are currently limited, with the underlying genetic aberration rarely explored [6]. To our knowledge, this is the first report of ADPKD complicated with aortic dissection caused by *PKD2* mutation.

Clinical course of ADPKD patients varies depending on the mutated gene. Patients caused by *PKD2* mutations have a milder form of disease, with a slower decline of renal function and onset at an older age [15]. Consistent with the observation, patients with ADPKD in our family have a normal renal function. ADPKD is a heterogeneous monogenic disorder with considerable intra-and interfamily phenotypic diversity. Even with the same mutation, the presence of vascular phenotype was highly variable as illustrated in our study, suggesting modifier effects of additional genetic or environmental factors [16]. Indeed, ADPKD patients are prone to develop hypertension via multiple mechanism, and hypertension in turn could cause tear in already vulnerable aortic walls. In accordance, hypertension has been reported to be more prevalent in ADPKD-associated aortic dissection patients. Long-term hypertension may therefore serve as superimposed risk factor that could modify the propensity toward dissection formation in ADPKD. With respect to its molecular diagnosis, sequencing of the candidate genes is currently not part of the standard care attributed in part to the difficulty of the practice. Conventional Sanger sequencing is hampered by coding sequence rich in GC nucleotides and six highly homogenous pseudogenes of *PKD1* [17]. In recent years, next-generation sequencing has evolved as a robust method to detect mutations in ADPKD patients [18]. With an elaborate design of the capture strategy and improved sequencing depth, WES can achieve an excellent diagnostic effect. In this study, we successfully identified the culprit mutation by employing WES.

In conclusion, we identified a stop-gain mutation of *PKD2* in an ADPKD family complicated with asymptomatic aortic dissection. Our report summarizes the phenotype concerning the deadly vascular complication. This report illustrates the importance of aorta imaging in ADPKD patients with uncontrolled hypertension and WES could serve as a robust method to detect the underlying mutation.

## Abbreviations

ADPKD: Autosomal dominant polycystic kidney disease
BWA: Burrows-wheeler aligner
ESRD: End-stage renal disease
GATK: Genome analysis toolkit
Indels: Insertions and deletions
IRAD: International registry of acute aortic dissection
SNVs: Single nucleotide variants
WES: Whole exome sequencing
